# The Impact of Separation Anxiety and Psychological Resilience on Parental Attitudes in Individuals Who Attended Boarding School During Adolescence

**DOI:** 10.1192/j.eurpsy.2025.1295

**Published:** 2025-08-26

**Authors:** T. Sarikavak, N. Elkin

**Affiliations:** 1 Psychiatry, Istanbul Atlas University; 2 Psychiatry, Humanite Medical Center; 3 Child Development, Istanbul Gelisim University, Istanbul, Türkiye

## Abstract

**Introduction:**

Boarding schools, seen across various cultures and regions, aim to broaden access to quality education, especially for disadvantaged groups. These schools, operated by different institutions, offer distinct educational approaches. State-affiliated boarding schools centralize resources to provide conditions not easily available elsewhere, fostering independence and discipline. Families often send their children to boarding schools, hoping they will gain opportunities to improve their lives and possibly benefit the family as a whole. However, early separation from family can have both positive and negative long-term effects on children’s socio-emotional development.

**Objectives:**

One of the potential outcomes is undoubtedly separation anxiety, which may arise from early separation from the family. On the other hand, this separation might also result in an increase in psychological resilience, as it forces individuals to live independently at an early age. In this study, we aimed to explore how separation anxiety and psychological resilience are impacted by this experience, and how these effects influence the parental attitudes that individuals adopt when raising their own children.

**Methods:**

The study was designed as a case-control study, and participants were recruited through snowball sampling. Data were collected either through face-to-face interviews or online surveys. To gather data, a Sociodemographic Data Form, which included personal and educational information, along with the DSM-5 Separation Anxiety Disorder Severity Scale-Adult Form, the Resilience Scale for Adults (RSA) and Parentıng Attıtude Scale (PAS) were used. After examining whether the data were normally distributed based on Skewness and Kurtosis values, Student’s t-test, Mann-Whitney U, and Chi-square tests were used for group comparisons. Relationships between variables were analyzed using Pearson and Spearman correlation tests, as well as Stepwise Regression analysis.

**Results:**

A total of 107 volunteers who attended boarding school during adolescence participated in this study. Of these, 41 were male (38%), and 39 (36%) attended boarding school during high school. The average total score for separation anxiety was 0.890 for those who attended boarding school and 0.675 for those who did not.

**Conclusions:**

This study has allowed us to gain a deeper understanding of the impact of an educational and social phenomenon, such as attending boarding school, on mental health. It is valuable in demonstrating the effects of established social institutions on individual mental health, and to the best of our knowledge, it is among the first studies of its kind. The examination of its impact on parental attitudes also sheds light on the intergenerational transmission of these changes.

**Disclosure of Interest:**

None Declared

